# Comparison between radiological and nasopharyngolaryngoscopic assessment of adenoid tissue volume in mouth breathing children

**DOI:** 10.1016/S1808-8694(15)31280-5

**Published:** 2015-10-20

**Authors:** Edmir Américo Lourenço, Karen de Carvalho Lopes, Álvaro Pontes, Marcelo Henrique de Oliveira, Adriana Umemura, Ana Laura Vargas

**Affiliations:** 1 Joint Professor, Ph.D., Professor responsible for the Discipline of Otorhinolaryngology, Medical School, Jundiaí; 2 Resident Physician, Discipline of Otorhinolaryngology, Medical School, Jundiaí; 3 Resident Physician, Discipline of Otorhinolaryngology, Medical School, Jundiaí; 4 Resident Physician, Discipline of Otorhinolaryngology, Medical School, Jundiaí; 5 Resident Physician, Discipline of Otorhinolaryngology, Medical School, Jundiaí; 6 Resident Physician, Discipline of Otorhinolaryngology, Medical School, Jundiaí

**Keywords:** adenoid tissue, mouth breathing, radiology of nasopharynx, nasopharyngolaryngoscopy

## Abstract

The pharyngeal tonsil (adenoid) constitutes the upper portion of the Waldeyer's ring and is located at the top of the nasopharynx, next to the auditory tube and choana. It plays an important role in recurrent otitis of the middle ear and many times its enlargement is responsible for upper airway obstruction. Tonsillectomy is often the treatment of choice for tonsillar diseases. So far, it is the most frequent and one of the oldest surgical procedures performed in children and young adults. The criteria for tonsillectomy, its effect on patient's immunological integrity and the surgical risks are widely controversial. Image study using paranasal sinuses x-ray is a very simple, easy and comfortable method to evaluate the sizes of adenoids and the grade of upper airway obstruction. Cohen et al. supported that paranasal sinuses x-ray is the best way to determine pharyngeal tonsil hypertrophy. On the other hand, nasopharyngolaryngoscopy can provide more accurate data on the nasopharynx, as it can dynamically reveal its structures and the obstruction status of the upper airway. This study compared the grade of adenoid hypertrophy, as well as upper airway obstruction, using the above-mentioned approaches in children ranging from 3 to 10 years old. The study came to the conclusion that nasopharyngolaryngoscopy is a much more accurate diagnostic procedure than radiological evaluation of the nasopharynx.

## INTRODUCTION

The pharyngeal tonsil, also called adenoid, is the upper extension of the lymphatic Waldeyer's Ring and is located on the upper posterior wall of the nasopharynx^1^. It is found adjacently to the choanae and the auditory tube ostium. Adenoid hypertrophy plays an important role in recurrent otitis as well as in secreting otitis of the middle ear. Many times, this structure is associated with enlargement of palatine tonsil, which leads to obstruction of upper airways and may host chronic recurrent pharyngeal infections^2^.

Adenoidectomy and/or tonsillectomy are surgical approaches frequently adopted in Otolaryngology, and are among the oldest surgeries to which human beings have been submitted in the past years. Recently, emphasis over careful selection of prospects for these procedures emerged from a consensus on the immunological role played by palatine and pharyngeal tonsils, as well as the potential complications of these types of surgery^3^.

Lateral x-ray of facial sinuses, including soft tissues for paranasal sinuses visualization, is an accessible procedure for the physician and relatively comfortable for the child^4^, consisting of a simple way to determine adenoids' size, shape and position^3^. Cohen et al. agree that this is an appropriate modality to evaluate children with suspected adenoidal hypertrophy^5^. On the other hand, flexible nasofibroscopy is an endoscopic method that allows direct visualization of the nasopharynx, including the auditory tube and fossa of Rosenmuller, action of the velopharyngeal sphincter and, consequently, functional evaluation of this region^6^. Some authors emphasize that, for a comprehensive sight of nasopharynx, flexible nasofibroscopy must be followed by an x-ray, providing reliable data on the relationship between content and continent^7^.

This study aims at evaluating and comparing the grade of adenoidal hypertrophy through simple radiological evaluation of the paranasal sinuses profile and endoscopy assessment by flexible nasofibroscopy in mouth breathing children.

## MATERIAL AND METHOD

Twenty (20) mouth breathing children, ages ranging from 3 to 10 years, were randomly selected at the Ambulatory of Otolaryngology of Medical School, Jundiaí, Hospital das Clínicas of Franco da Rocha, Sao Paulo – SP (DIR-IV) in the period of March and June 2004.

Children's parents or caregivers signed a Term of Consent to whom explanations about the study were given. Furthermore, a questionnaire was filled out with information about symptoms, such as presence of snoring, nocturnal drooling, noisy sleep, mouth breathing, nocturnal sialorrhea, daytime sleepiness, nose itching, sneezing, hyaline rhinorrhea, and nasal obstruction. Following, an otolaryngological examination was performed to evaluate hypertrophy and coloration of nasal conchae, presence and aspect of rhinorrhea and grading of palatine tonsil hypertrophy.

Only children who were not under medication or inflammatory/infectious process of the airways were selected for the study.

A simple x-ray of the paranasal sinuses profile was performed to which the patient was asked to inhale, in standing position and with the mouth shut; these instructions were given both to the x-ray professional and the child's parent/caregiver. Immediately after that, the child was submitted to flexible nasofibroscopy. For that, local anesthesia was applied on nasal fossas with 10% lidocaine and oximetazoline with vasoconstrictor, locally. During examination, as soon as full visualization of the choana was reached, the patient was asked for deep nostril inhaling, as to obtain a reliable image regarding the true obstruction of choanal opening.

**Figure 1:**
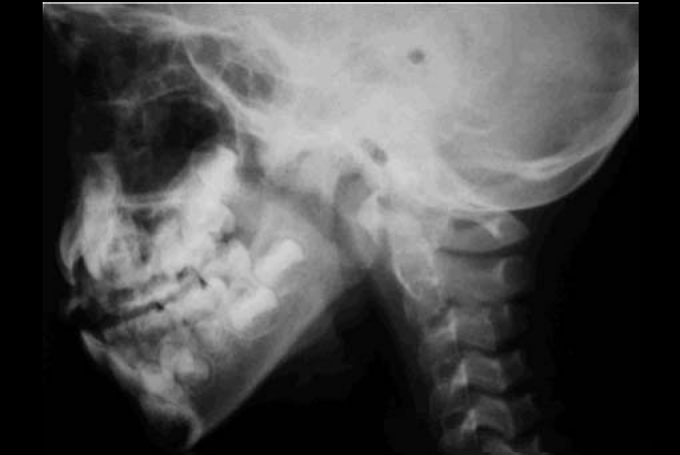
Plain x-ray paranasal sinuses profile.

**Figure 2:**
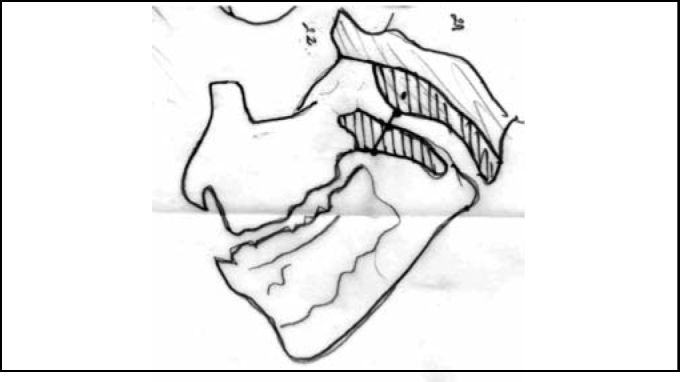
Scheme illustrating Cohen & Konak method, which compares the soft palate thickness (one centimeter below the hard palate or half centimeter in children younger 3 years old) presenting the air column between this spot in the palate and the highest convexity spot of adenoid (blue line). In the present example, it is a large adenoid.
2) All nasofibroscopic procedures were initially videotaped (VHS), among which the best choanal images were selected and printed by Videoprinter Sony® (Figure 3). After manually outlining the choanal and adenoid limits, these photos were scanned by Corel Scan 7.0 software, posteriorly processed by Corel Photo Paint 7.0 in bitmap files (Figure 4) and analyzed by Corel Trace 7.0 as vector figures (Figure 5). Through this software, it was possible to assess, with decimal accuracy, the area occupied by the choanal adenoid.

Assessment of methodology criteria included:

1) Interpretation of x-ray paranasal sinuses profile was based on Cohen & Konak^8^ method in which the soft palate thickness (one centimeter below the hard palate or half-centimeter in children younger than 3 years) and the air column width between the palate and the highest point of convexity of the adenoid are compared. It is considered small when the column is not narrower than the palate's thickness; medium, when air column is narrower, but wider than half of the palate's thickness; large, when the air column is narrower that half of palate's thickness (Figures 1 and 2).

**Figure 3:**
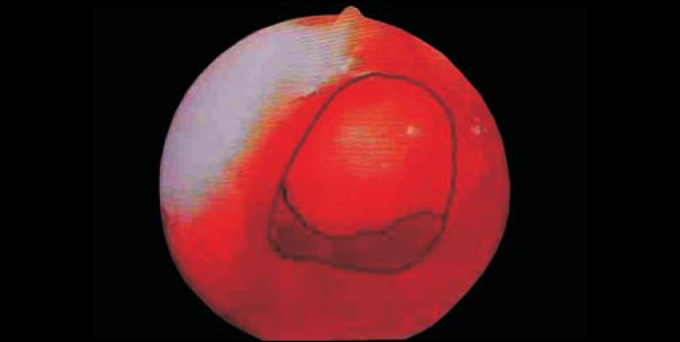
Sample of a selected image printed by Videoprinter Sony®.

**Figure 4:**
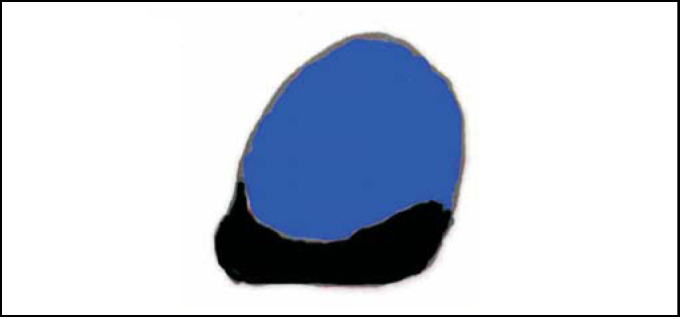
Sample of a scanned image by Corel Scan 7.0, further processed by Corel Photo Paint 7.0 in bitmap file.

**Figure 5:**
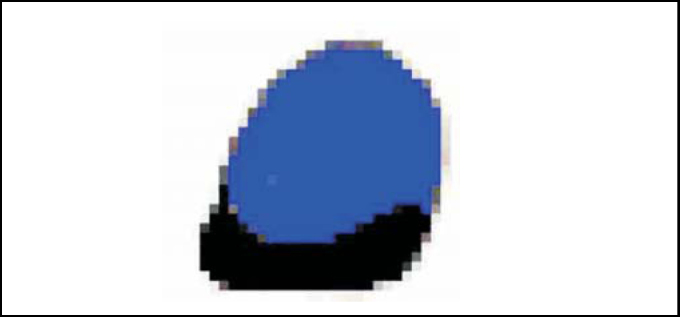
Sample of image analyzed by Corel Trace 7.0 as a vector figure.

For better understanding, it was considered small adenoid when it occupied less than half of choana; medium adenoid, around 50 and 70% of the choana; and large adenoid, when occupying over 75% of full choanal area. Interpretations of both evaluations were independent and not correlated with the history data and clinical findings.

**RESULTS**

## DISCUSSION

The first adenoidectomy was probably performed in the second half of the 19^th^ Century. For a long time, due to inexistence of clear criteria for indication of surgery, this procedure fell into disbelief among physicians and public opinion. Recently, accurate indications and clear rules for adenoidectomy have proved to be less controversial^2^. The literature reports a concern regarding the best way to diagnose and treat children with suspected adenoid hypertrophy, a very frequent condition observed in Otorhinolaryngology. Clinical evaluation of adenoid size in young children is very difficult. History reported by parents of nasal obstruction, mouth breathing, nocturnal drooling and speech disorders ground the relation with adenoid enlargement, not visible at direct inspection through anterior rhinoscopy and oroscopy; regarding posterior rhinoscopy, besides the technical difficulty in approaching young children, its real value is controversial^9^. Objective measures of adenoid hypertrophy are useful to provide information that may help deciding the need of surgery and subsequent outcomes' evaluation.

Today, there is not much consensus over the best way of checking the size and position of adenoid tissue in preoperative evaluation. Mignon formerly observed the shadow of adenoidal tissue in 1898. Later, it was verified that this tissue narrowed the nasopharynx and, after that, many authors investigated different aspects of adenoid and nasopharynx x-rays in an attempt to minimize chances of misinterpretation^10^. There are reports of different radiographic methods for evaluation of nasopharynx, while the interpretation of presence or absence of adenoid hypertrophy is not a consensus among authors. According to Wormald et al. who conducted a comparative study among methods, Cohen & Konak developed the best approach providing the highest positive predictive value^11^. According to these authors, their method takes into account the relation between nasopharynx and adenoid sizes, besides being a simple approach, once it does not require measures and calculations. Moreover, they emphasize that the otolaryngologist should consider the air column rather than adenoid's size or shape, leading to physiological interpretation^8^. Cohen, Konai and Scott support the idea that lateral x-ray of nasopharynx is an effective method to evaluate children with suspected adenoid hypertrophy^5^; however, x-rays have some disadvantages, as they consist of irradiation on the child, not mentioning the lack of standardization in technique and film evaluation, besides the two-dimensional image of nasopharynx rather than a three-dimensional structure.

Hirschmann^6^ firstly performed a nasosinusal endoscopy in 1901. Technical improvement of otolaryngological endoscopy was gradual. Initially, this procedure was limited only to a few professionals, but it was gradually incorporated into medical practices as improved optical and more comfortable instruments - both to the patient and the examiner - were developed. Currently, diagnostic nasofibroscopy is an important complementary exam to assess patients with complaint of nasal obstruction.

So far, there are no reports of standardization for nasopharynx endoscopy evaluation. This way, methods using imaginary lines and reference spots^7^ up to examiners' subjective evaluations are verified. The assessment method developed in this study outstands for being an objective, easy and reproducible approach. Wormald et al. report that, in doubtful cases, nasal endoscopy under local anesthesia provides a definitive evaluation of the nasal cavity and nasopharynx state^11^. Another study consistent with our findings showed that nasofibroscopy is more reliable than lateral x-ray of the paranasal sinuses to assess size and shape of pharyngeal tonsil^4^. Difficulty in submitting non-collaborative young children to endoscopy is a disadvantageous feature of this procedure^5^, ^6^; however, in our sample, this situation occurred with patients under 4 years old.

According to Table 1 and based on Cohen & Konak method, out of 20 children submitted to paranasal sinuses x-rays, 7 presented small, 6 medium and 7 large adenoids. Furthermore, out of 20 children submitted to nasofibroscopy, 5 presented medium and 15 large adenoids. There were definitely no children with small adenoid structure revealed by nasofibroscopy and this is undoubtedly a relevant finding. According to Table 1, a numerical score was determined for each size of adenoid in increasing levels, in which 1 point was credit to small adenoids, 2 points for medium adenoids, and 3 points for large adenoids (Table 2). This way, it was possible to determine the arithmetical mean of adenoid sizes through paranasal sinuses x-ray and nasofibroscopy. Average x-rayed size of adenoids in a 1-3 scale was 2.0, while through nasofibroscopy it was 2.75. Therefore, adenoids evaluated by nasofibroscopy were on average 37.5% larger as compared to adenoids evaluated by paranasal sinuses x-ray. Percentage mean of choanal obstruction showed by nasofibroscopy was 79.5%, which corresponds to an average volume of a large adenoid - considered as greater than 75% in this study.

**Table 1:** Individual scoring of adenoid size through x-ray and nasofibroscopy; % - percentage of choanal obstruction.

Radiological assessment (Cohen & Konak Method)		Nasofibroscopy assessment		
X-Ray	Classification	Photo	%	Classification
1	Medium	1	69	Medium
2	Medium	2	82	Large
3	Large	3	80	Large
4	Small	4	87	Large
5	Small	5	67	Medium
6	Medium	6	83	Large
7	Small	7	73	Medium
8	Small	8	75	Medium
9	Small	9	86	Large
10	Small	10	87	Large
11	Large	11	87	Large
12	Large	12	87	Large
13	Medium	13	80	Large
14	Large	14	82	Large
15	Large	15	83	Large
16	Small	16	56	Medium
17	Medium	17	87	Large
18	Medium	18	76	Large
19	Large	19	78	Large
20	Large	20	85	Large

**Table 2:** Scoring by adenoid size: 1 = small, 2 = medium, 3 = large.

X-Ray	Radiological Evaluation	Photo	Nasofibroscopy Evaluation
1	2	1	2
2	2	2	3
3	3	3	3
4	1	4	3
5	1	5	2
6	2	6	3
7	1	7	2
8	1	8	2
9	1	9	3
10	1	10	3
11	3	11	3
12	3	12	3
13	2	13	3
14	3	14	3
15	3	15	3
16	1	16	2
17	2	17	3
18	2	18	3
19	3	19	3
20	3	20	3
Mean	2.00	Medium	2.75

**Graph 1:**
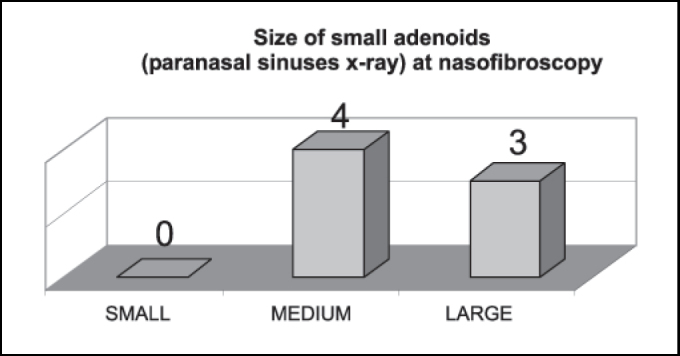
Correspondence of number of patients with radiologically small adenoids (n = 7) at nasofibroscopy.

Graph 1 shows that small adenoids by paranasal sinuses x-ray were 100% considered medium or large structures by nasofibroscopy.

**Graph 2:**
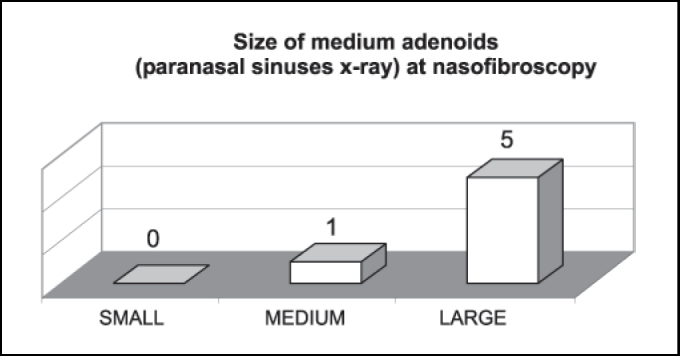
Correspondence of number of patients with radiologically medium adenoids (n = 6) at nasofibroscopy.

Graph 2 shows that medium adenoids by paranasal sinuses x-ray were mostly considered large structures by nasofibroscopy.

**Graph 3:**
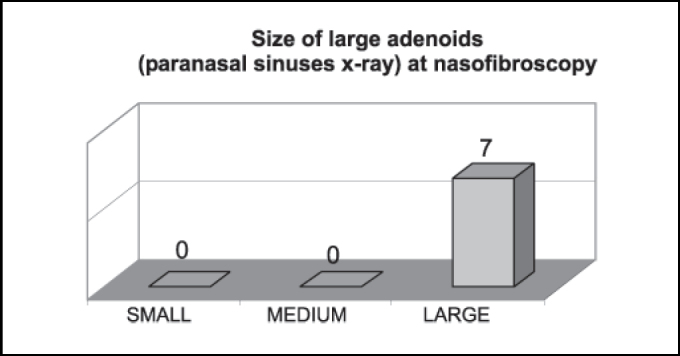
Correspondence of number of patients with radiologically large adenoids (n = 7) at nasofibroscopy.

Graph 3 shows that large adenoids by paranasal sinuses x-ray were considered large structures by nasofibroscopy.

In Graph 4, it was observed that 100% of children presented snoring and mouth breathing, 85% presented nocturnal sialorrhea and 45% daytime sleepiness.

Based on the above findings, it is possible to verify that children with classical symptoms of major respiratory obstruction, even without adenoid hypertrophy revealed by x-ray, should be submitted to nasofibroscopy for diagnostic accuracy, which is greatly relevant, specially for a more secure indication for adenoidectomy.

## CLOSING REMARKS

For assessment of adenoidal enlargement, nasofibroscopy is a diagnostic approach by far more reliable than paranasal sinuses x-ray.
